# First Insights into Changes in the Gut Microbiota of Landes Geese Infected with *Eimeria stigmosa*

**DOI:** 10.3390/ani16162470

**Published:** 2026-08-08

**Authors:** Ya-Qi Bu, Shuo Li, Zi-Rui Wang, Qian Liu, Xing-Quan Zhu, Qing Liu

**Affiliations:** Shanxi Key Laboratory of Animal Disease Research, Prevention and Control, College of Veterinary Medicine, Shanxi Agricultural University, Jinzhong 030801, China; 15832277876@163.com (Y.-Q.B.); leenianzi@163.com (S.L.); wangzirui8091@126.com (Z.-R.W.); l15525635339@163.com (Q.L.); xingquanzhu1@hotmail.com (X.-Q.Z.)

**Keywords:** *Eimeria stigmosa*, geese, 16S ribosomal RNA gene sequencing, jejunal and cecal microbiota

## Abstract

While multiple *Eimeria* species have been identified in geese, their effects on the host gut microbiota have not yet been investigated. In the present study, 16S ribosomal RNA gene sequencing was used to characterize the microbial changes in the jejunal and cecal segments of Landes geese following *Eimeria stigmosa* infection. At 2.5 days post-infection (dpi), the jejunum of infected geese was dominated by Bacillota, Pseudomonadota and Bacteroidota, whereas controls were dominated by Bacillota and Actinomycetota. At 4.5 dpi, the jejunum of infected geese showed increased Bacillota compared to controls. In the cecum, both groups shared similar phylum-level dominance of Bacillota and Bacteroidota. At 2.5 and 4.5 dpi, the jejunal microbiota of infected geese was primarily dominated by the genus *Ligilactobacillus*. α-diversity analysis revealed a significant reduction in the Shannon and Pielou’s evenness indices only in the jejunum of infected geese at 4.5 dpi. Beta-diversity analysis showed significant differences between the infected and control groups at 2.5 dpi based on weighted UniFrac distances. LEfSe analysis revealed that the genus *Pseudomonas* were significantly enriched in the jejunum of infected geese at 2.5 dpi, while the genera *Massiliomicrobiota* and *Shuttleworthia* were significantly enriched in the cecum of infected geese at 4.5 dpi. These findings revealed time- and site-specific shifts in the gut microbiota of geese following *E. stigmosa* infection, having implications for better understanding of the complex interactions of *E. stigmosa*, host and the microbiome.

## Simple Summary

Though several studies have examined how chicken *Eimeria* species affect gut microbiota, the effects of goose-infecting *Eimeria* species on the host gut microbiota remain unexplored. Herein, we used 16S ribosomal RNA gene sequencing to investigate changes in the gut microbiota of landes geese following *Eimeria stigmosa* infection. Regarding the jejunal microbiota, *E. stigmosa* infection caused clear phylum-level shifts at 2.5 and 4.5 dpi. The jejunal microbiota in the infected group was primarily dominated by the genus *Ligilactobacillus* at 2.5 and 4.5 dpi. Regarding the cecal microbiota, similar community structures were observed between the infected and healthy geese. α-diversity analysis revealed a significant reduction in the Shannon and Pielou’s evenness indices only in the jejunal microbiota of infected geese at 4.5 dpi, while β-diversity analysis showed significant differences in the jejunal microbiota between the infected and control groups at 2.5 dpi. LEfSe analysis identified significant enrichment of *Pseudomonas* in the jejunum of infected geese at 2.5 dpi, while the genera *Massiliomicrobiota* and *Shuttleworthia* were significantly enriched in the cecum of infected geese at 4.5 dpi. These observations advance our understanding of the complex interactions of *E. stigmosa*, host and the microbiome.

## 1. Introduction

*Eimeria* parasites are ubiquitous pathogens infecting a wide range of avian species and exhibiting species specificity [1]. Regarding poultry, chickens are known to host seven long-recognized *Eimeria* species, alongside three recently proposed ones [2,3]. Several of these species are both highly virulent and commonly found, causing enormous economic damage to the poultry industry [4,5,6,7]. Meanwhile, goose farming is also an important sector of the poultry industry. To date, several *Eimeria* species have been identified in geese, such as *Eimeria stigmosa*, *Eimeria truncata*, *Eimeria nocens*, *Eimeria magnalabia*, *Eimeria anseris*, and *Eimeria hermani* [8,9,10]. Of which, *E. truncata* is a highly pathogenic species, and its life cycle exhibits common and distinct features compared with that of *Eimeria* species infecting chickens [10]. For example, a distinct feature is that *E. truncata* parasitizes the kidneys of geese [10].

Also, the life cycle of *E. stigmosa* in geese displays common and distinct features compared with that of *Eimeria* species infecting chickens. Asexual and sexual developmental forms of *E. stigmosa* were found in the jejunum and ileum of the small intestine [10]. Additionally, sexual stages were also present in the caecum and colon [11]. Regarding asexual stages of *E. stigmosa*, schizonts were present from 1.5 to 4 days post-infection (dpi), with mature forms peaking at 2 and 3 dpi [11]. Regarding sexual stages of *E. stigmosa*, gametocytes were present at 4 and 5 dpi [12].

The severity of *Eimeria* infection in geese is species-dependent, and *E. stigmosa* does not cause obvious clinical disease or death [10]. Apart from directly causing adverse effects on the host, several *Eimeria* species were reported to be able to alter gut microbial composition [13,14,15]. Gut microbiota play critical roles in maintaining host health, such as helping to build the intestinal epithelial barrier and supporting the development and function of the host immune system [16,17]. To date, however, no information currently exists regarding the impact of *E. stigmosa* infection on the gut microbiota of geese. In the present study, we performed 16S ribosomal RNA gene sequencing (16S rRNA-seq) to characterize the microbial community in the jejunum and cecum of Landes geese following *E. stigmosa* infection. According to the life cycle characteristics of *E. stigmosa* mentioned above, 2.5 (representing the asexual stage) and 4.5 dpi (representing the sexual stage) were selected as the sampling time points.

## 2. Materials and Methods

### 2.1. Parasites and Animals

Sporulated oocysts of *E. stigmosa* wild type Shanxi strain (SX-01) (isolated and preserved in the Laboratory of Parasitic Diseases, College of Veterinary Medicine, Shanxi Agricultural University) were used for animal experiments. One-day-old Landes geese were purchased from a commercial hatchery and reared under coccidia-free conditions. Throughout the duration of the experiment, feed and water free of anticoccidials and antibiotics were provided ad libitum.

### 2.2. Experimental Design and Sample Collection

Twenty-one-day-old male Landes geese were randomly divided into an infected (I) group and a control (C) group, with six geese in each group. Immediately after grouping, each goose in the I group was orally infected with a dose of 1.5 × 10^5^ *E. stigmosa* sporulated oocysts suspended in 1 mL PBS, while each goose in the C group received 1 mL of PBS. Three geese per group were euthanized via cervical dislocation at 2.5 and 4.5 dpi. Following dissection of the intestine, jejunal contents (400 mg) and jejunal mucosa samples were collected at 2.5 and 4.5 dpi. Cecal contents (400 mg) cecal mucosa samples were collected only at 4.5 dpi, given that the cecum is not the site of parasitism at 2.5 dpi and that this study focused on the effects of *E. stigmosa* parasitism on the gut microbiota at the parasitic site. Meanwhile, each sample was placed into a sterile 1.5 mL plastic tube, immediately frozen in liquid nitrogen upon collection, and subsequently kept at −80 °C.

### 2.3. DNA Extraction, PCR Amplification, and Sequencing

To confirm the successful establishment of *E. stigmosa* infection, total DNA was separately extracted from cecal and jejunal mucosa samples using a blood/cell/tissue genome DNA extraction kit (Tiangen Biotech Co., Beijing, China) according to the manufacturer’s instructions. Each DNA sample was subjected to PCR amplification of the 18S rRNA gene fragment using primers described previously [18].

Total DNA was separately extracted from cecal and jejunal contents using a TIANamp Stool DNA Kit (Tiangen Biotech Co., Beijing, China) following a previous study [19]. The concentration and purity of the extracted DNA were determined by running it on a 1% agarose gel. Qualified DNA samples were then diluted with sterile nuclease-free water to achieve a final concentration of 1 ng/μL.

Then, extracted DNA was used as a template to amplify the V3–V4 region of the bacterial 16S rRNA gene using the barcode-fusion forward primer 341F (5′-barcode-CCTAYGGGRBGCASCAG-3′) and the reverse primer 806R (5′-GGACTACNNGGGTATCTAAT-3′) [19]. Each PCR reaction mixture contained 15 µL of Phusion High-Fidelity PCR Master Mix (New England Biolabs, Ipswich, MA, USA), 0.2 µM of each primer, and 10 ng of genomic DNA template. The thermal cycling program consisted of an initial denaturation step at 98 °C for 1 min, followed by 30 cycles of denaturation at 98 °C lasting 10 s, a primer annealing step at 50 °C for 30 s, and extension at 72 °C for 30 s; after that, a final extension was carried at the end of the run at 72 °C for 5 min. The PCR amplicons were purified using magnetic beads for library construction, and sequencing was carried out on an Illumina NovaSeq 6000 platform by Novogene Co., Ltd. (Beijing, China).

### 2.4. Data Analysis for Bacterial 16S rRNA Sequencing

Based on unique barcode sequences and PCR amplification primer sequences, demultiplexing of raw sequencing data was performed. After the barcode and primer sequences had been removed, the resulting paired-end reads were subjected to assembly using the FLASH software (Version 1.2.11) (Center for Computational Biology, Johns Hopkins University, Baltimore, MD, USA) [20], and then trimmed using Cutadapt software (Version 3.3) (National Bioinformatics Infrastructure Sweden, Uppsala, Sweden). To obtain high-quality clean tags, the raw tags were filtered using fastp software (Version 0.23.1) (HaploX Biotechnology, Shenzhen, China) [21]. PCR chimera detection and removal (using a reference based approach) was performed by comparison with the reference database (Silva database, https://www.arbsilva.de/, accessed on 11 July 2024) using vsearch software (Version 2.16.0, University of Oslo, Oslo, Norway), yielding the effective Tags. Quality filtering, denoising, de novo chimera removal, and singleton removal were performed using the DADA2 plugin in QIIME2 (Version 202504, Caporaso Lab, Northern Arizona University, Flagstaff, AZ, USA) to obtain amplicon sequence variants (ASVs). Subsequent analyses (including species annotation, alpha-diversity analysis, and beta-diversity analysis) were conducted using QIIME2 (Version 202504) and R software (Version 4.0.3, R Core Team). To calculate alpha diversity, four metrics were calculated: the bias-corrected Chao1 estimator, Simpson index, Shannon diversity index, and Pielou’s evenness. The Benjamini-Hochberg method was used to control the false discovery rate (FDR), with adjusted *p*-values (*q*-values) < 0.05 considered statistically significant. Beta diversity analysis was carried out using the weighted and unweighted UniFrac distance methods. Also. The Benjamini–Hochberg method was used to control the false discovery rate (FDR), with adjusted *p*-values (*q*-values) < 0.05 considered statistically significant. Beta diversity analysis was carried out using the weighted and unweighted UniFrac distance methods. Differentially abundant taxa were identified by linear discriminant analysis (LDA) effect size (LEfSe) analysis, which integrates the Kruskal–Wallis test, Wilcoxon rank-sum test, and LDA, with a significance threshold of *p* < 0.05 and an LDA score cutoff of 2.0.

## 3. Results

### 3.1. Characteristics of 16S rRNA-Seq Reads

No obvious signs of infection were observed in geese from any group. PCR results confirmed the successful establishment of *E. stigmosa* infection in the infected group, as the expected DNA band was amplified from infected samples but not from controls (Figure S1). To investigate the impact of *E. stigmosa* infection on microbial community composition and diversity in the jejunum and cecum of geese, high-throughput 16S rRNA-seq analysis was performed. Sequencing data are summarized in Table 1. In total, 1,249,841 raw reads were generated for the jejunum; of which, 1,233,451 effective tags were obtained after quality filtering. Meanwhile, 605,456 raw reads were generated for the cecum; of which, 494,920 effective tags were obtained after quality filtering. For all samples, the Q20 value exceeded 98.5%, while the Q30 value exceeded 95%; the average sequence length ranged from 411.03 to 428.94. These indicated that the 16S rDNA sequencing results was of excellent quality.

### 3.2. Shared and Unique Microbial Populations

**Figure 1:**
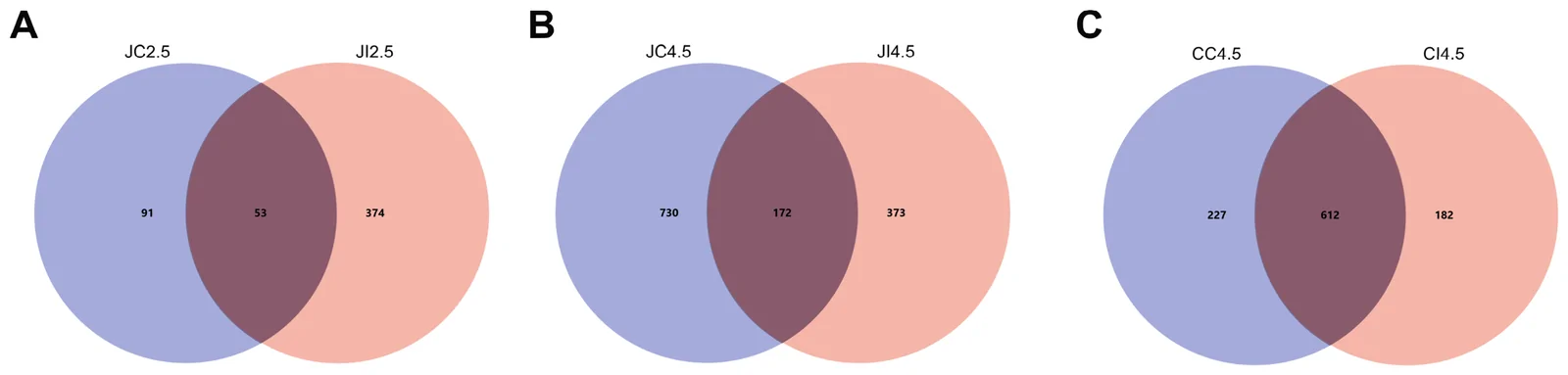
Venn diagrams were used to show the unique and shared number of ASVs. (**A**) Venn diagram showing the number of ASVs in the jejunal contents of the infected and control groups at 2.5 dpi. JC = the jejunum of uninfected geese; JI = the jejunum of *Eimeria stigmosa*-infected geese. (**B**) Venn diagram showing the number of ASVs in the jejunal contents of the infected and control groups at 4.5 dpi. (**C**) Venn diagram showing the number of ASVs in the cecal contents of the infected and control groups. CC = the cecum of uninfected geese; CI = the cecum of *E. stigmosa*-infected geese.

To investigate the microbial community following *E. stigmosa* infection, we examined the unique and shared ASVs between the infected group and control groups in the goose cecum at 4.5 dpi and in the jejunum at both 2.5 and 4.5 dpi. In the jejunum at 2.5 dpi, 53 ASVs were shared between the infected and control groups, whereas 374 and 91 unique ASVs were detected in the infected and control groups, respectively (Figure 1A). In the jejunum at 4.5 dpi, 172 ASVs were shared between the infected and control groups, whereas 373 and 730 unique ASVs were detected in the infected and control groups, respectively (Figure 1B). In the cecum, a total of 1021 ASVs were detected. Of which, 612 were shared between the control and infected groups, while 227 and 182 unique ASVs were observed in the control and infected groups, respectively (Figure 1C).

### 3.3. Analysis of α-Diversity

**Figure 2:**
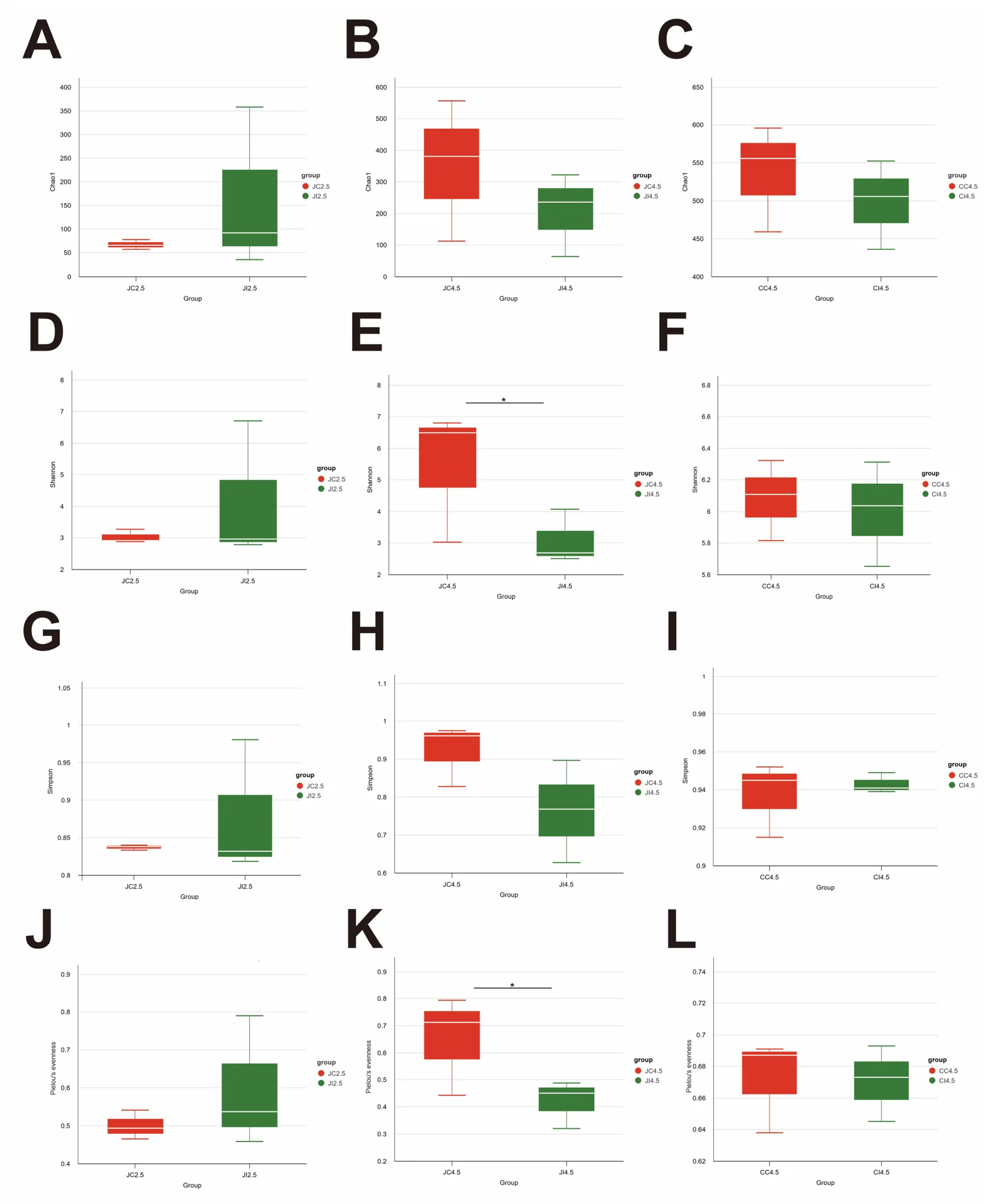
(**A**–**C**) Assessing species richness by the Chao1 index. (**D**–**F**) Assessing species richness by the Shannon index. (**G**–**I**) Assessing species richness by the Simpson index. (**J**–**L**) Pielou’s species evenness were calculated. JC = the jejunum of uninfected geese; JI = the jejunum of *Eimeria stigmosa*-infected geese; CC = the cecum of uninfected geese; CI = the cecum of *E. stigmosa*-infected geese. “*” indicates statistically significant (adjust *p*-value <  0.05).

α-diversity metrics were used to reveal the richness and diversity of the jejunal and cecal microbiota, including the Chao1, Shannon, Simpson indices, and Pielou’s evenness. The Chao1, Shannon, Simpson, and Pielou’s evenness indices did not differ significantly between the two groups in the jejunum at 2.5 dpi or in the cecum at 4.5 dpi (Figure 2). However, in the jejunum at 4.5 dpi, the Shannon diversity and Pielou’s evenness indices were significantly lower in the infected group than in the control group (Figure 2).

### 3.4. Analysis of β-Diversity

**Figure 3:**
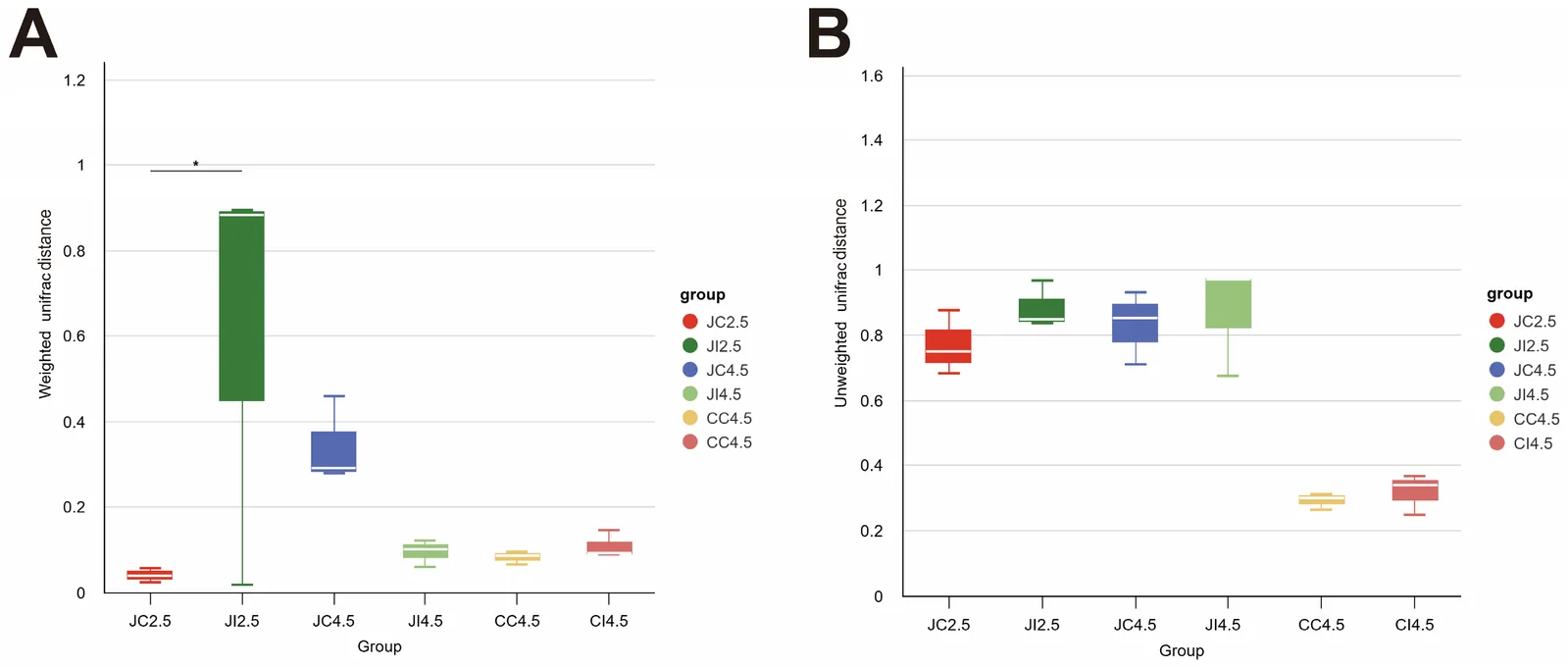
A box plot of beta diversity based on the weighted and unweighted UniFrac distance calculations. The Kruskal–Wallis test was used to analyze the differences between the weighted (**A**) and unweighted (**B**) distances among groups. JC = the jejunum of uninfected geese; JI = the jejunum of *Eimeria stigmosa*-infected geese; CC = the cecum of uninfected geese; CI = the cecum of *E. stigmosa*-infected geese. “*” indicates statistically significant (adjust *p*-value <  0.05).

At 2.5 dpi, β-diversity analysis showed that a significant difference was detected between the infected group and the control group using the weighted UniFrac distance matrix but not for unweighted UnifFrac (Figure 3). At 4.5 dpi, the infected and control groups exhibited no significant differences in β-diversity within the jejunal and cecal microbiota.

### 3.5. Analysis of Microbial Composition and Abundance in the Jejunum and Cecum of Geese

**Figure 4:**
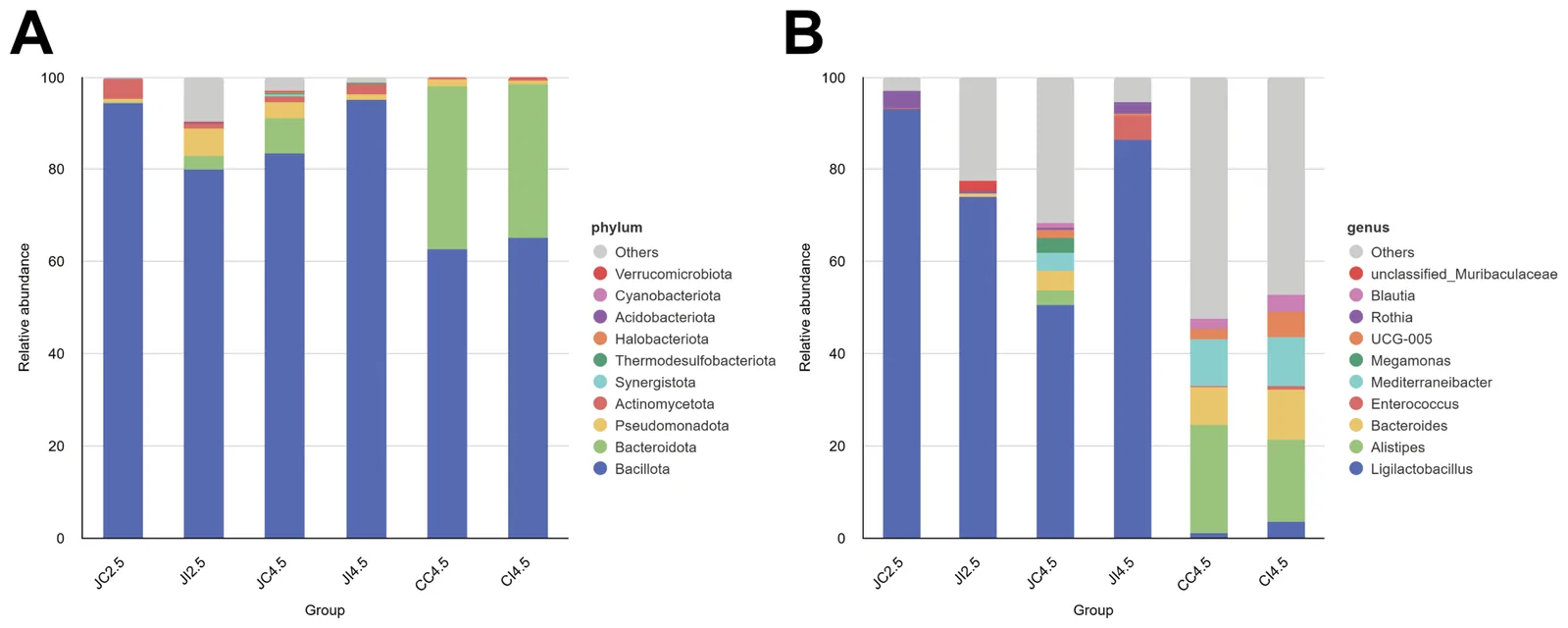
The gut microbiota structure of the jejunum and cecum at the phylum (**A**) and genus (**B**) level among the various sample groups. JC = the jejunum of uninfected geese; JI = the jejunum of *Eimeria stigmosa*-infected geese; CC = the cecum of uninfected geese; CI = the cecum of *E. stigmosa*-infected geese.

At 2.5 dpi, analysis of the jejunal microbiota at the phylum level revealed that Bacillota (80.05%), Pseudomonadota (5.88%), and Bacteroidota (2.85%) were found to be the most abundant in the infected group. In the control group, Bacillota (94.48%) and Actinomycetota (4.24%) were the most abundant (Figure 4A). At the genus level, *Ligilactobacillus* (74.12%), unclassified_*Muribaculaceae* (2.22%), and *Acinetobacter* (2.05%) were found to be the most abundant in the infected group, while *Ligilactobacillus* (93.22%) and *Rothia* (3.60%) were the most abundant in the control group (Figure 4B).

At 4.5 dpi, analysis of the jejunal microbiota at the phylum level revealed that Bacillota (95.02%) and Actinomycetota (2.49%) were found to be the most abundant in the infected group. In the control group, Bacillota (83.47%), Bacteroidota (7.78%), and Pseudomonadota (3.40%) were the most abundant (Figure 4A). At the genus level, *Ligilactobacillus* (86.44%), *Enterococcus* (5.21%), and *Rothia* (2.35%) were found to be the most abundant in the infected group, while *Ligilactobacillus* (50.68%), *Bacteroides* (4.13%), *Mediterraneibacter* (3.92%), *Megamonas* (3.41%), and *Alistipes* (3.15%) were the most abundant in the control group (Figure 4B).

At 4.5 dpi, analysis of the cecal microbiota at the phylum level revealed that Bacillota (65.24%) and Bacteroidota (33.31%) were the two most predominant in the infected group. In the control group, Bacillota (62.77%) and Bacteroidota (35.21%) were also the most abundant (Figure 4A). At the genus level, *Alistipes* (17.61%), *Bacteroides* (10.99%), *Mediterraneibacter* (10.68%), and *Ligilactobacillus* (3.51%) were found to be the most abundant in the infected dpi group, while *Alistipes* (23.06%), *Mediterraneibacter* (10.05%), *Bacteroides* (8.53%), and *Ligilactobacillus* (1.10%) were the most abundant in the control group (Figure 4B).

### 3.6. Differential Microbial Analysis Using LEfSe

**Figure 5:**
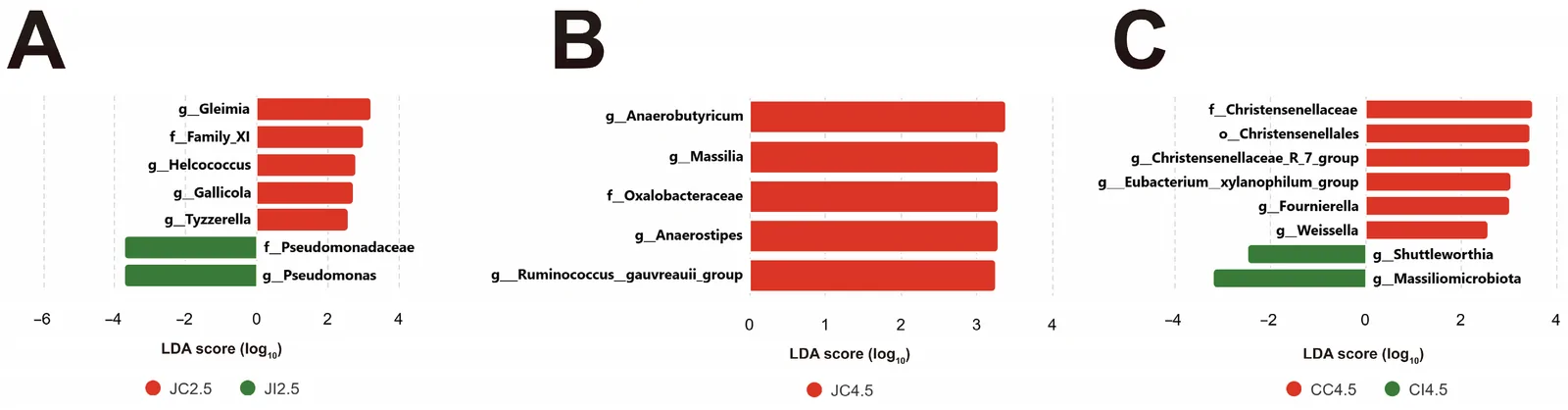
Differential enrichment of jejunal and cecal bacteria. (**A**) LEFSe analysis indicated differences in the bacterial groups identified in the jejunal microbiota of infected and control geese at 2.5 dpi. (**B**) LEFSe analysis indicated differences in the bacterial groups identified in the jejunal microbiota of infected and control geese at 4.5 dpi. (**C**) LEFSe analysis indicated differences in the bacterial groups identified in the cecal microbiota of infected and control geese. JC = the jejunum of uninfected geese; JI = the jejunum of *Eimeria stigmosa*-infected geese; CC = the cecum of uninfected geese; CI = the cecum of *E. stigmosa*-infected geese.

In the jejunum at 2.5 dpi, further analysis using LEfSe revealed that the relative abundance of the genus *Pseudomonas* increased significantly in the infected group compared with the control group (Figure 5A). In the jejunum at 4.5 dpi, no bacteria were significantly increased in the infected group compared with the control group (Figure 5B). In the cecum, analysis using LEfSe revealed that the relative abundance of the genera *Massiliomicrobiota* and *Shuttleworthia* increased significantly in the infected group compared with the control group (Figure 5C).

## 4. Discussion

Gut microbiota, the most important active components in the intestinal microecosystem, play important roles in host health, including immunity, nutrition, and protection from pathogens [22,23]. In the present study, high-throughput 16S rDNA amplicon sequencing was carried out to reveal changes in the gut microbiota of geese following *E. stigmosa* infection. The sequencing quality was high and the data were reliable, as indicated by a Q30 value exceeding 95% and an appropriate average sequence length. In addition, the cecal microbial composition (including Bacillota, Bacteroidota, Pseudomonadota, Actinomycetota, Synergistota, Thermodesulfobacteriota, Acidobacteriota, Cyanobacteriota, and Verrucomicrobiota) was similar to that reported in a previous study [24]. This further indicated that the microbial composition was accurately captured.

The Shannon index is an indicator of species richness and evenness, while Chao1 is a diversity index used to reflect species richness [25,26]. In the present study, the Shannon index of the jejunal microbiota was markedly lower in the infected group when compared with the control group at 4.5 dpi. Meanwhile, no significant differences in Chao1 diversity were observed between the infected and control groups in the jejunal microbiota at 4.5 dpi. This indicated that *E. stigmosa* infection altered species evenness in the jejunal microbiota at 4.5 dpi, which was further confirmed by a significant difference in Pielou’s diversity. Also, a previous study showed that evenness of the gut microbiota significantly decreased during *Eimeria maxima* infection [27]. Although no significant differences in alpha diversity were observed in the jejunal microbiota between the infected group compared with the control group at 2.5 dpi, a significant difference was detected in β-diversity based on the weighted UniFrac distance matrix but not the unweighted UniFrac matrix. This indicated that there was inter-group heterogeneity in community structures despite comparable species richness and evenness. Both UniFrac distances account for phylogenetic branch length; the weighted UniFrac distance accounts for both the phylogenetic relationships and the relative abundances of taxa, whereas the unweighted UniFrac distance reflects differences in microbial presence/absence [28]. This indicated that similar taxa were present in the infected group and the control group at 2.5 dpi, but there were differences in the relative abundances of the taxa.

In the present study, *Ligilactobacillus* was found to be the dominant genus in the jejunum of both infected and uninfected geese at 2.5 and 4.5 dpi. A decrease in the relative abundance of *Ligilactobacillus* was observed in the control group, falling from 93% at 2.5 dpi to 51% at 4.5 dpi, a trend consistent with previous observations in chicken jejunum over time [14]. Meanwhile, an increase from 74% to 86% was detected over time in the infected group in the present study, a pattern similar to that observed in *Eimeria tenella*-infected chickens [14]. Notably, the variation was much higher in the control group than in the infected group. A previous study showed that the reduced levels of intestinal proinflammatory cytokines and elevated levels of tight junction proteins were correlated with the increased abundance of *Ligilactobacillus* [29]. Hence, it is possible that *E. stigmosa* infection may induce a slight decrease in *Ligilactobacillus* abundance, but the host may subsequently elevate its level, thereby improving intestinal barrier integrity and decreasing pro-inflammatory cytokine levels.

LEfSe analysis of the 16S rRNA sequencing data showed that the abundance of the genus *Pseudomonas* in the jejunum of infected geese was higher in the infected group compared with the control group at 2.5 dpi. The genus *Pseudomonas* includes many pathogens, affecting intestinal health [30,31]. In the cecum, LDA scores showed that the genus *Shuttleworthia* was overrepresented in the infected group compared with the control group at 4.5 dpi. The genus *Shuttleworthia* was known for their roles in producing short-chain fatty acid (SCFA), which preserves intestinal barrier integrity [32,33]. This indicated that the genus *Shuttleworthia* may play important roles in supporting cecal health during *E. stigmosa* infection.

Of course, there were limitations in the present study. One was that we tested only three samples per group, which met the minimal requirement for biological replicates. In addition, only one goose *Eimeria* species was included in the present study. More studies involving various goose *Eimeria* species are warranted to better understand the complex interactions between goose *Eimeria* species, the host, and the intestinal microbiome.

## 5. Conclusions

The effects of *E. stigmosa* infection on microbial diversity, composition, and enrichment varied depending on the time point and intestinal segment examined. In terms of diversity, α-diversity declined significantly only in the jejunum of infected geese at 4.5 dpi, while β-diversity revealed distinct clustering between infected and control groups at 2.5 dpi. Regarding compositional changes, *E. stigmosa* infection caused clear phylum-level shifts at 2.5 and 4.5 dpi, whereas the cecum remained relatively stable at the phylum level; at the genus level, *Ligilactobacillus* consistently dominated the jejunal microbiota at both time points. The relative abundance of the genus *Pseudomonas* increased significantly in the jejunum of infected geese at 2.5 dpi, while the genus *Shuttleworthia* was enriched in the cecum of infected geese at 4.5 dpi. These findings provide a foundational understanding of the complex interactions between *E. stigmosa*, the host, and the intestinal microbiome.

## Figures and Tables

**Table 1:** Summary of sequencing data. JC = the jejunum of uninfected geese; JI = the jejunum of *Eimeria stigmosa*-infected geese; CC = the cecum of uninfected geese; CI = the cecum of *E. stigmosa*-infected geese.

Sample Name	Raw Reads	Clean Tags	Effective Tags	Average Length	Q20	Q30	GC%
JC2.5-1	106,112	106,084	105,977	427.06	98.75%	96.08%	49.37%
JC2.5-2	105,520	105,493	104,707	428.14	98.67%	95.81%	48.85%
JC2.5-3	98,603	98,581	98,551	428.82	98.55%	95.36%	48.69%
JI2.5-1	104,927	103,350	103,126	415.56	98.92%	96.64%	51.36%
JI2.5-2	89,969	89,952	89,897	428.94	98.63%	95.68%	48.58%
JI2.5-3	105,600	105,573	105,475	428.73	98.67%	95.79%	48.69%
JC4.5-1	106,339	106,052	101,526	417.53	99.05%	97.02%	51.82%
JC4.5-2	105,170	105,109	101,776	416.07	99.00%	96.81%	51.17%
JC4.5-3	103,185	103,167	102,792	428.54	98.50%	95.30%	48.76%
JI4.5-1	115,499	115,483	115,224	428.78	98.52%	95.31%	48.67%
JI4.5-2	104,738	104,711	101,091	426.55	98.91%	96.54%	49.91%
JI4.5-3	104,179	103,852	103,309	427.48	98.78%	96.11%	48.98%
CC4.5-1	105,996	105,982	81,252	413.73	99.24%	97.49%	51.25%
CC4.5-2	104,220	104,206	97,903	412.89	99.16%	97.25%	51.44%
CC4.5-3	81,937	81,928	70,541	411.85	99.22%	97.45%	51.49%
CI4.5-1	102,896	102,888	75,829	415.78	99.22%	97.45%	51.15%
CI4.5-2	105,525	105,514	79,798	411.03	99.26%	97.59%	51.84%
CI4.5-3	104,882	104,868	89,597	411.73	99.23%	97.47%	51.46%

## Data Availability

Processed data supporting the findings of this study are available from the corresponding author on reasonable request. The raw reads generated by 16S rRNA amplicon sequencing have been deposited in the NCBI Sequence Read Archive (SRA) under BioProject accession number PRJNA1489804.

## Supplementary Materials

The following supporting information can be downloaded at https://www.mdpi.com/article/10.3390/ani16162470/s1. Figure S1: The successful establishment of *E. stigmosa* infection in the inoculated group was confirmed by PCR analysis. (A) DNA extracted from jejunal samples collected at 2.5 dpi. (B) DNA extracted from jejunal samples collected at 4.5 dpi. (C) DNA extracted from cecal samples collected at 4.5 dpi. Lane M = DNA marker. JC = the jejunum of uninfected geese; JI = the jejunum of *Eimeria stigmosa*-infected geese; CC = the cecum of uninfected geese; CI = the cecum of *E. stigmosa*-infected geese.
