# Supplementary material for: First Insights into Changes in the Gut Microbiota of Landes Geese Infected with Eimeria stigmosa
Source: Animals (Basel). 2026 Aug 8;16(16):2470. doi: 10.3390/ani16162470 (PMC13508863; doi:10.3390/ani16162470)
Supplement: Supplementary file 1 [file animals-16-02470-s001.zip › animals-4445800-supplementary.pdf]

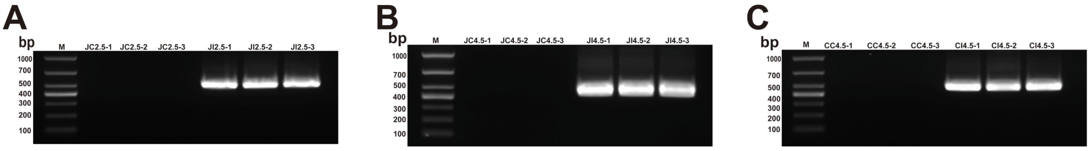

Figure S1. The successful establishment of *E. stigmosa* infection in the inoculated group was confirmed by PCR analysis. (A) DNA extracted from jejunal samples collected at 2.5 dpi. (B) DNA extracted from jejunal samples collected at 4.5 dpi. (C) DNA extracted from cecal samples collected at 4.5 dpi. Lane M = DNA marker. JC = the jejunum of uninfected geese; JI = the jejunum of *Eimeria stigmosa*-infected geese; CC = the cecum of uninfected geese; CI = the cecum of *E. stigmosa*-infected geese.
